# MicroRNAs in Palatogenesis and Cleft Palate

**DOI:** 10.3389/fphys.2017.00165

**Published:** 2017-04-04

**Authors:** Christian Schoen, Armaz Aschrafi, Michelle Thonissen, Geert Poelmans, Johannes W. Von den Hoff, Carine E. L. Carels

**Affiliations:** ^1^Department of Orthodontics and Craniofacial Biology, Radboud University Medical CenterNijmegen, Netherlands; ^2^Laboratory of Molecular Biology, Division of Intramural Research Programs, National Institute of Mental Health, National Institutes of HealthBethesda, MD, USA; ^3^Department of Human Genetics, Radboud University Medical CenterNijmegen, Netherlands; ^4^Department of Cognitive Neuroscience, Donders Institute for Brain, Cognition and Behaviour, Radboud University Medical CenterNijmegen, Netherlands; ^5^Department of Molecular Animal Physiology, Donders Institute for Brain, Cognition and Behaviour, Radboud Institute for Molecular Life Sciences, Radboud UniversityNijmegen, Netherlands; ^6^Department of Oral Health Sciences, University Hospitals—KU LeuvenLeuven, Belgium

**Keywords:** miRNA, palatogenesis, cleft palate, post-transcriptional regulation, genetics

## Abstract

Palatogenesis requires a precise spatiotemporal regulation of gene expression, which is controlled by an intricate network of transcription factors and their corresponding DNA motifs. Even minor perturbations of this network may cause cleft palate, the most common congenital craniofacial defect in humans. MicroRNAs (miRNAs), a class of small regulatory non-coding RNAs, have elicited strong interest as key regulators of embryological development, and as etiological factors in disease. MiRNAs function as post-transcriptional repressors of gene expression and are therefore able to fine-tune gene regulatory networks. Several miRNAs are already identified to be involved in congenital diseases. Recent evidence from research in zebrafish and mice indicates that miRNAs are key factors in both normal palatogenesis and cleft palate formation. Here, we provide an overview of recently identified molecular mechanisms underlying palatogenesis involving specific miRNAs, and discuss how dysregulation of these miRNAs may result in cleft palate.

## Introduction

Cleft palate represents the most common craniofacial birth defect, occurring on its own, in combination with a cleft lip or as part of a genetic syndrome (Mossey and Modell, 2012). It represents a significant healthcare burden requiring multidisciplinary treatment starting shortly after birth up to adulthood. Although many syndromic and non-syndromic familial forms of cleft palate have been described, approximately 70% of all cases are isolated non-syndromic entities without clear Mendelian inheritance patterns (Dixon et al., 2011). These non-syndromic forms have a complex etiology caused by both genomic and environmental factors and their interactions. Through candidate gene and genome-wide association studies (GWASs), a number of protein-coding susceptibility genes for cleft palate have been identified (Mangold et al., 2011). Unfortunately, the protein-coding genes identified thus far only account for a small fraction of the total genetic risk associated with cleft palate (Khandelwal et al., 2013). Moreover, when analyzing GWAS data, more than 80% of disease-associated genetic loci are found outside protein-coding genes (Manolio et al., 2009), indicating an important role for the non-coding genome in the etiology of cleft palate.

**Figure 1:**
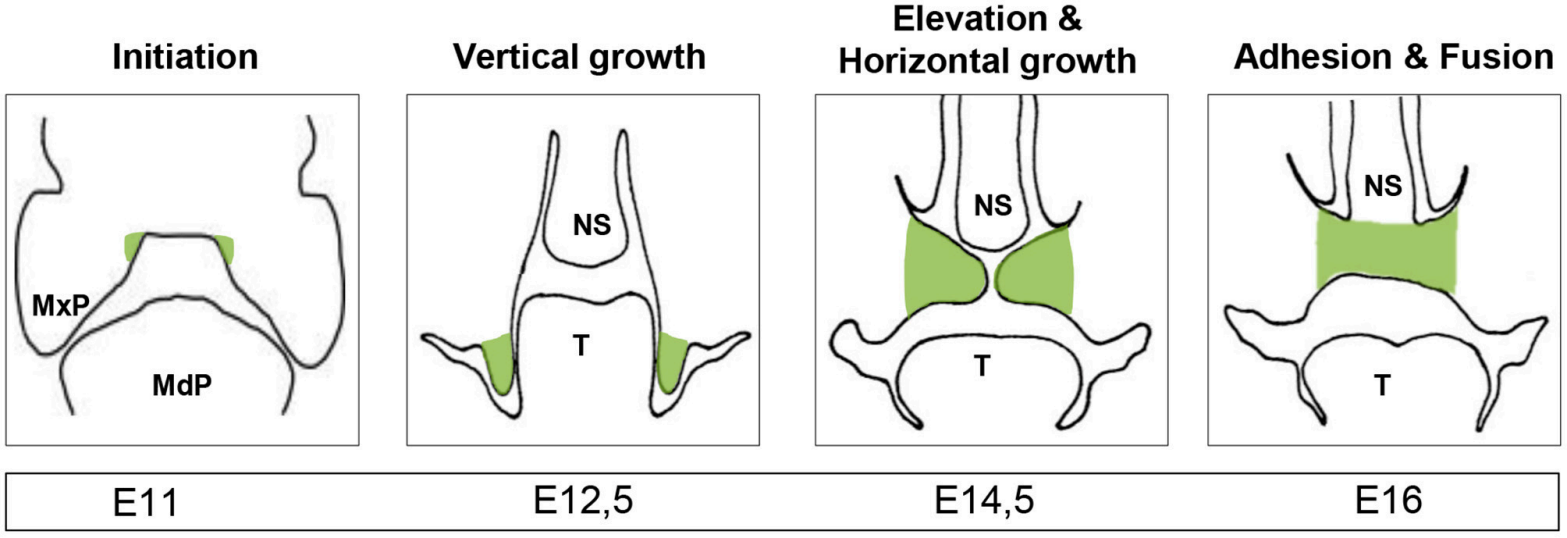
**Palatogenesis**. Illustration of the developing secondary palate (highlighted in green) through frontal sections of a mouse embryo with the timing of the relevant stages below. MxP, maxillary process; MdP, mandibular process; NS, nasal septum; T, tongue; highlighted in green: palatal shelves.

The formation of the secondary palate requires a tightly regulated sequence of events spanning weeks 6 to 12 of human gestation and E11 to E16 in mice (Figure 1). Perturbations in any of these events can lead to an impaired fusion of the bilateral palatal shelves (PS) and hence to cleft palate (Bush and Jiang, 2012). The first step consists of cranial neural crest cells migrating to the maxillary process of the first pharyngeal arch where reciprocal signaling with the oral ectoderm drives outgrowth of the PS (Bush and Jiang, 2012). Outgrowth is followed by elevation and fusion of both shelves with eventual disintegration of the midline epithelial seam (Meng et al., 2009). Specific signaling pathways and gene expression patterns control each step of palatal fusion and are extensively reviewed elsewhere (Meng et al., 2009; Bush and Jiang, 2012). As these events are tightly regulated, it is understandable that many transcriptional regulators have been identified as key etiological factors in cleft palate (Beaty et al., 2016). Recent studies by the ENCODE consortium indicate that certain non-coding regions of the genome, chromosomal arrangements and nuclear domains are also key regulators of gene expression (Consortium et al., 2012). As such, alterations in the non-coding genome—including non-coding RNAs, and particularly miRNAs (Pauli et al., 2011)—are able to change gene expression during embryogenesis. In the present review, we provide an overview of the emerging concepts on the roles of miRNAs during palatogenesis and cleft palate.

### MiRNAs as regulators of embryonic development and disease

**Figure 2:**
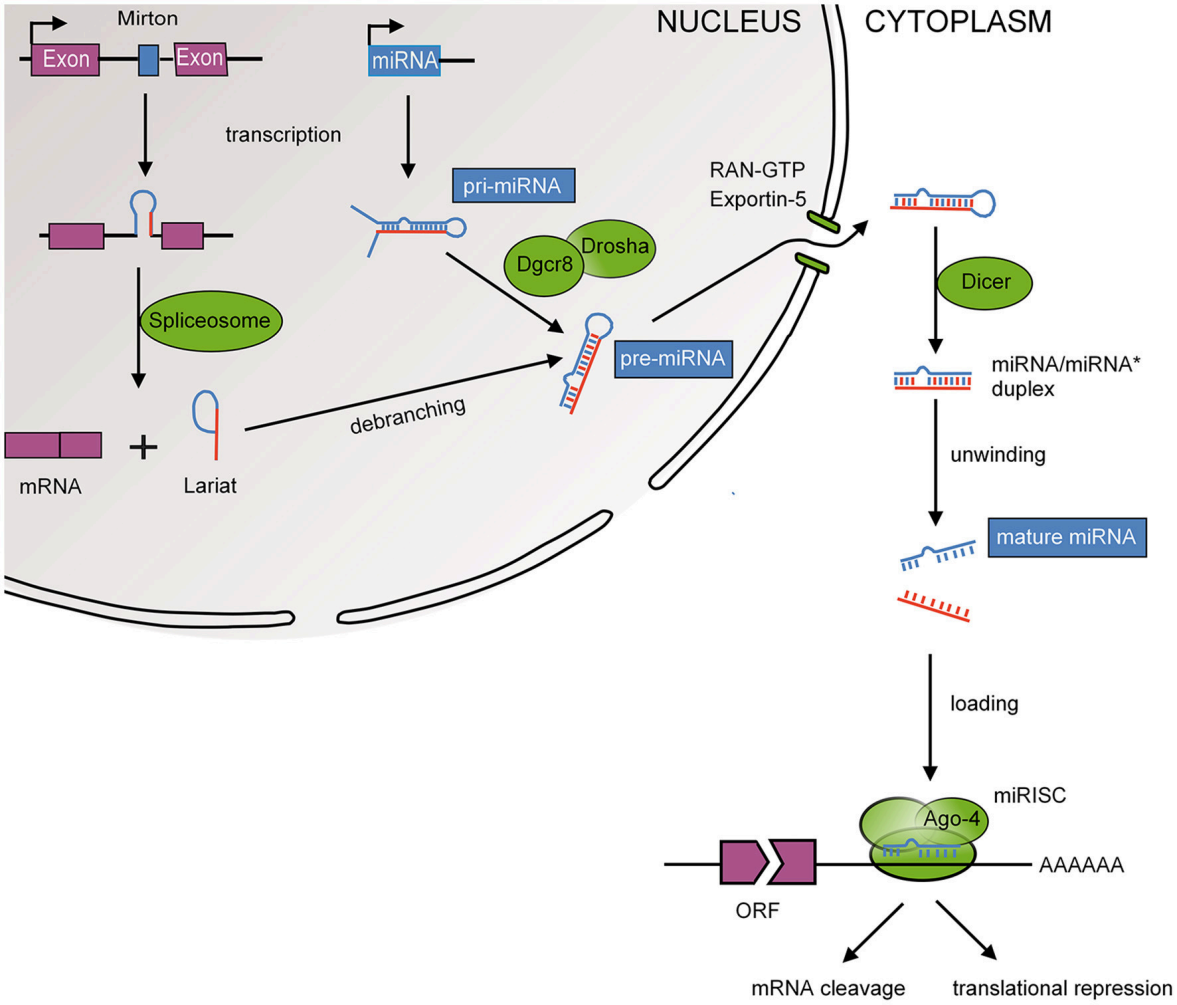
**MiRNA biogenesis**. Mature miRNAs are encoded in the genome and form after a series of enzymatic cleavages from two possible precursor molecules; primary miRNAs (pri-miRNAs) or Mirtrons. Pri-miRNAs, following the canonical pathway, are transcribed as long hairpin RNAs that are recognized by the RNA-binding DiGeorge syndrome critical region 8 protein (DGCR8). Many pri-miRNAs are often transcribed simultaneously due to clustering of several miRNA genes (Ambros et al., 2003). DGCR8 then directs the RNase III endonuclease DROSHA to cleave the base of the hairpin to produce ~70 nucleotide hairpins known as pre-miRNA. Mirtrons, following the non-canonical pathway, bypass the microprocessor as they are transcribed as part of the introns of protein coding genes and are as such spliced by the spliceosome (Berezikov et al., 2007). Splicing also produces ~70 nucleotide hairpins known as pre-miRNA. The pre-miRNA is transported to the cytoplasm by exportin 5 where it is cleaved by another RNase III endonuclease known as DICER to ~20 nucleotide miRNA duplexes with protruding 2 nucleotide 3′ ends. The resulting mature miRNA is released and a guiding strand is incorporated into the RNA-induced silencing complex (RISC).

MiRNAs are small, 19–23 nucleotide non-coding RNAs that function as post-transcriptional repressors of gene expression, either through messenger RNA (mRNA) degradation or translational repression (Bartel, 2009). The biogenesis and action of miRNAs is depicted in Figure 2. Translational repression is mediated by pairing of the 5′ region (seed region) of the miRNA to the 3′-untranslated region (UTR) of the mRNA within RNA-induced silencing complexes (RISCs). Important characteristics of miRNA-mediated repression are redundancy (one mRNA is targeted by many miRNAs) and multiplicity (one miRNA targets many mRNAs; Herschkowitz and Fu, 2011). This facilitates concurrent regulation of mRNAs that function in similar cellular processes and suggests that miRNAs have evolved into functionally related groups. The first miRNA was discovered in 1993, i.e., *Lin-4* in *C. elegans*, a miRNA that regulates larval patterning during development (Lee et al., 1993). Regulatory roles for RNAs had been postulated previously but were regarded as exceptions to the rule that transcription factors were the main regulators of gene expression (Britten and Davidson, 1969). At present, miRNAs have been implicated in a wide range of developmental processes including epithelial-mesenchymal transition (EMT), cell migration, differentiation, proliferation, and apoptosis (Mathieu and Ruohola-Baker, 2013). Hence, miRNAs are key regulators of embryogenesis (Pauli et al., 2011). As a reflection of these wide-ranging regulatory roles, the online miRNA database (miRBase 21; http://www.mirbase.org) contains more than 24,000 gene loci encoding more than 30,000 miRNAs in 193 species (Kozomara and Griffiths-Jones, 2014). Of these, ~2,000 miRNAs have been identified in the human genome.

**Figure 3:**
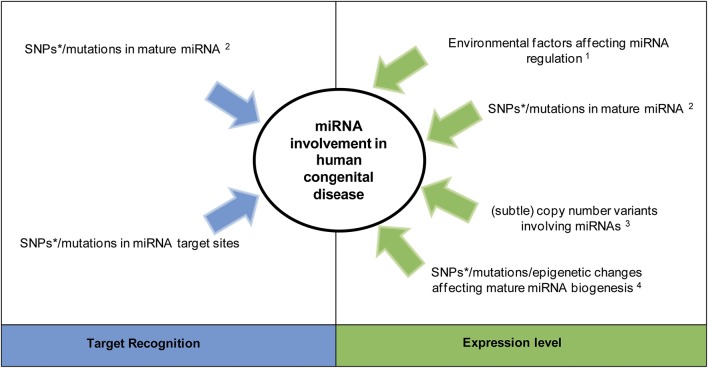
**MiRNA involvement in congenital disease**. Alterations affecting miRNA activity by changing target recognition or modulating their expression. ^*^Single nucleotide polymorphisms (SNPs) within miRNAs are likely involved in complex disease (common disease, common variant hypothesis). ^1^Environmental factors can directly or indirectly regulate miRNA expression independent of any germline miRNA alteration (Zhao et al., 2008). ^2^A SNP/mutation within the miRNA seed sequence can alter both its processing and target recognition, while a change outside the seed sequence only alters miRNA processing (Duan et al., 2007). ^3^ Large copy number variants lead to syndromes while subtle ones (those only detectable via molecular methods) are predicted to be involved in complex diseases (Shelling and Ferguson, 2007). ^4^Germline alterations of regulators belonging to one of the two miRNA biogenesis/ processing pathways (i.e., the pathways involving mature miRNA generation from pri-miRNAs or Mirtrons, see Figure 2) will only change the expression level of mature miRNAs being generated through this pathway (Finnegan and Pasquinelli, 2013). Epigenetic changes in this context refer to functional changes without a change in the DNA sequence, such as methylation and histone modification.

Recent evidence suggests an involvement of a set of specific miRNAs in the pathogenesis of certain congenital disorders with a chromosomal abnormality or monogenic cause (Kawahara, 2014). This occurs through germline alterations affecting either miRNA target recognition or expression level (Figure 3). The number of congenital disorders caused by a single defective miRNA are likely limited due to their redundancy (Meola et al., 2009). In *C. elegans*, only 10% of individual miRNA knockouts leads to a clear developmental defect (Miska et al., 2007). An alteration in the function of one miRNA may be (partially) compensated by other miRNAs, and hence not lead to a disease phenotype. However, altered expression profiles of several miRNAs have been identified in many complex, multifactorial diseases, for example cardiovascular diseases (van Rooij and Olson, 2007). As shown in Figure 3, changes in many similarly expressed miRNAs acting synergistically on disease-associated mRNAs may therefore contribute to common congenital diseases within a multifactorial model (Chavali et al., 2013).

### Global role of miRNAs in palatogenesis

**Figure 4:**
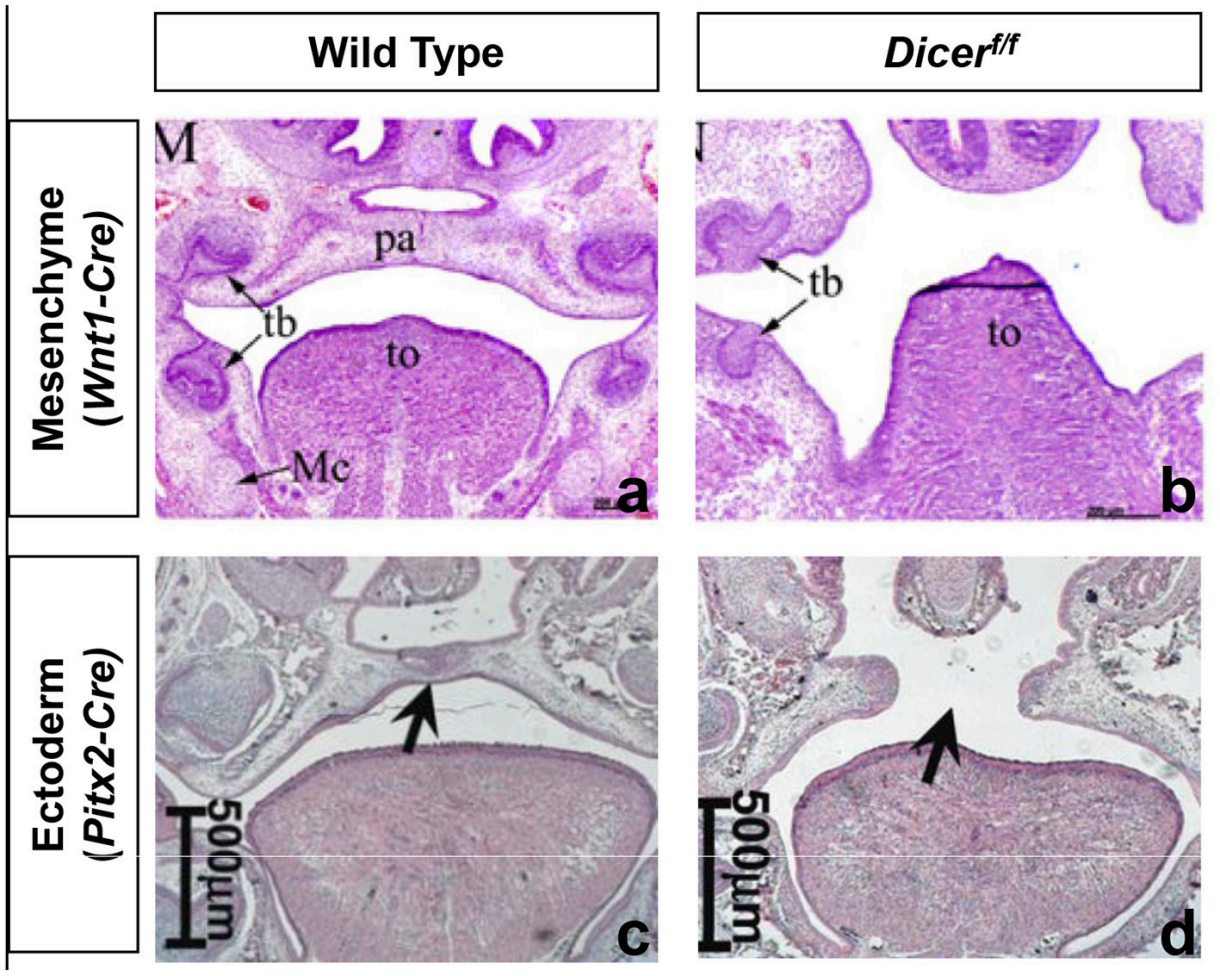
**Homozygous conditional deletion of Dicer in neural crest derived mesenchyme and oral ectoderm**. Coronal sections of E18.5 wild-type and *Wnt1-Cre; Dicer*^*f/f*^ **(a,b)** and E16.5 wild type and *Pitx2-Cre; Dicer*^*f/f*^ **(c,d)**. Black arrow: (left) palate. **(a,b)** Adapted from Nie et al. (2011). **(c,d)** Adapted from Cao et al. (2010). pa, palate; tb, tooth bud; Mc, Meckel's cartilage; To, tongue.

An initial approach to determine the role of miRNAs in vertebrate development has been to genetically delete *Dicer* and *Dgcr8* in mice. As these two proteins are required for the maturation of most miRNAs, their deletion will deplete most functional miRNAs (Graves and Zeng, 2012). Homozygous zygotic deletion of either gene in mice leads to severe growth retardation and embryonic lethality shortly after implantation (Bernstein et al., 2003; Wang et al., 2007). Additional studies showed that miRNAs have fundamentally different developmental roles depending on the tissue (Spruce et al., 2010). Conditional knockout (cKO) studies in mice of miRNAs in the cranial neural crest (cNC)-derived mesenchyme or oral ectoderm, have shown that miRNAs are essential for palatogenesis in mice (Figure 4).

Conditional deletion of *Dicer* controlled by *Pax2-Cre* or *Wnt1-Cre* leads to perinatal death with severe craniofacial malformations in mice (Sheehy et al., 2010; Zehir et al., 2010; Nie et al., 2011; Barritt et al., 2012). *Pax2* and *Wnt1* expression is specific for cNC-derived mesenchyme from embryonic day (E) 7.5 and 8.5, respectively. However, *Wnt1* is expressed in all cNC-derived tissues, while *Pax2* expression is limited to the first pharyngeal arch and the anterior skull. Therefore, a complete bilateral cleft palate develops in *Wnt1*-Cre; *Dicer*^*f*/*f*^ mice while primary palate development is not affected in the *Pax2-Cre; Dicer*^*f*/*f*^ mice. In both knockouts, a secondary palatal cleft develops due to absent vertical growth of the palatal shelves (PS). The epithelium overlying the hard palate has no significant histological changes, while that covering the soft palate is much thicker compared to controls (Otsuka-Tanaka et al., 2013).

The *Wnt1*-Cre; *Dicer*^*f*/*f*^ mice exhibited normal proliferation, migration and differentiation of cNC-derived mesenchyme but a large increase in apoptotic activity with lower levels of FGF8 and DLX2. Both are regulators of cNC-derived mesenchyme survival (Macatee et al., 2003; Dai et al., 2013). This may occur through miR-452, which is highly expressed in cNC-derived mesenchyme at E10.5 in the first pharyngeal arch and regulates Fgf8 and *Dlx2* expression. In the *Pax2-Cre; Dicer*^*f*/*f*^ mouse, a similarly large increase in apoptosis is present as well as a lower density of proliferating cells (Barritt et al., 2012). A *Wnt1*-Cre; *Dgcr8*^*f*/*f*^ mouse exhibited a phenotype similar to that of the *Dicer* deletion and showed lower levels of pERK1/2, a kinase involved in another cell survival pathway (Chapnik et al., 2012). This finding is particularly interesting because of the possible link with the human syndrome caused by a (heterozygous) microdeletion of chromosome region 22q11.2. This microdeletion syndrome has a large phenotypic variability with cleft palate as a common feature. Many genes lie within the deleted region, of which only *Tbx1* has been linked to cleft palate (Goudy et al., 2010; Funato et al., 2012; Gao et al., 2015). However, it requires a homozygous deletion of *Tbx1* for mice to develop a cleft palate, suggesting that other genes in the deleted 22q11.2 region—such as *Dgcr8*—might also contribute to the cleft palate observed with the syndrome (Herman et al., 2012). A link between TBX1 expression and miRNAs within the PS has already been established (Wang et al., 2013; Gao et al., 2015). It is also interesting to note that DGCR8 is strongly expressed in the developing PS of mice but further studies are needed to elucidate the role in normal palatogenesis and cleft palate formation (Shiohama et al., 2003). The data thus indicate that miRNAs are essential to maintain cNC-derived mesenchyme survival during the initial vertical outgrowth of the PS.

Conditional deletion of *Dicer* controlled by *Pitx2-Cre* and *Shh-Cre*—more specific for the oral ectoderm—leads to dental and palatal defects. By using the promoter of *Pitx2*, a gene that is expressed in the oral ectoderm as early as E10.5, for the conditional deletion of *Dice*r, a cleft palate with incomplete penetrance develops, in addition to several dental defects (Cao et al., 2010). Conditional *Dicer* deletion using the promoter of *Shh*, which is expressed as early as E10.5, leads to similar dental defects but no perturbed palatogenesis was reported (Oommen et al., 2012). These data indicate that, although the absence of miRNAs within the oral ectoderm of the developing palate can lead to cleft palate, it does not have a 100% penetrant effect.

### miRNA expression and function during palatogenesis

#### Expression during palatogenesis

Using microarray analysis, the expression profile of murine miRNAs in the developing lip and PS were analyzed from E10 to E14 (Mukhopadhyay et al., 2010; Warner et al., 2014). Most of the identified miRNAs exhibited a linear expression pattern over time and, for the PS, could be grouped into 6 specific patterns. Several miRNAs were expressed differentially in the PS, medial nasal process and maxillary process. Furthermore, 42 miRNA genes were found to be stably methylated within the PS (Seelan et al., 2014). These data suggest a specific and regulated spatiotemporal pattern of miRNAs may be crucial for palatogenesis. Apart from miR-140, the miR-17-92 cluster, and miR-200b (see below), most of the miRNAs have an as yet unknown role in palatogenesis. By focusing on a limited number of these unknown miRNAs, the authors demonstrated that many mRNAs important to palatogenesis are experimentally validated targets and that the miRNAs could be integrated in gene networks regulating processes such as cell proliferation, adhesion, apoptosis and EMT. In the developing lip, for instance, both miR-203 and members of the miR-302/367 cluster target different isoforms of *p63*, of which a deletion leads to cleft lip and palate (Warner et al., 2014). An additional expression study in mice, using small RNA sequencing, showed similar differential expression patterns over time (Ding et al., 2016). However, several additional miRNAs were identified including miR-23b and miR-133b. Over expression of both these miRNAs in zebrafish leads to broadening and a cleft, respectively, of the ethmoid plate, a component of the palatal skeleton in zebrafish.

Two avian studies identified several miRNAs in the developing frontonasal process with similar expression to that in mice (Darnell et al., 2006; Powder et al., 2012), suggesting an evolutionary conserved function. This may reflect the similar molecular mechanisms during early palatogenesis. However, in birds, the palatal shelves never fuse completely into a secondary palate and many of the identified miRNAs were avian-specific. While transcription/signaling pathways are largely conserved during evolution, miRNAs have been constantly added or lost and it has been hypothesized that they contribute to the increased complexity in higher vertrebrates (Heimberg et al., 2008). It is therefore interesting that avian-specific miRNAs were identified in the frontonasal process, but it remains to be investigated whether they have any functional role.

#### MiR-140 as regulator of cranial neural crest (cNC) migration

During neural tube closure, cNC cells delaminate from the neural fold and migrate in three streams toward the pharyngeal arches. Within the first pharyngeal arch the cNC cells fill the space adjacent to the oral ectoderm and undergo epithelial-mesenchymal interactions resulting in the vertical growth of the PS. Proper migration of cNC cells to the first pharyngeal arch is thus essential for palatogenesis. Studies in zebrafish have shown that proper miR-140 expression in migrating cNC cells is needed during palatogenesis. MiR-140 is a highly conserved miRNA that is located in an orthologous intron of *Wwp2*, which encodes a ubiquitin ligase that is essential for palatogenesis (Nakamura et al., 2011). In mice it has been identified that the transcription of both miR-140 and *Wwp2* is regulated by the SOX9 transcription factor (Nakamura et al., 2011, 2012). Interestingly, miR-140 also has its own regulatory element for SOX9, which suggest that its expression could be regulated independently of *Wwp2*. MiR-140 is broadly expressed in migrating cNC cells and gradually becomes restricted to skeletogenic crest cells, including those of the PS (Eberhart et al., 2008; Li et al., 2011). Within the PS, expression increases from E12 to E13 after which it levels off (Mukhopadhyay et al., 2010; Li et al., 2011).

MiR-140 overexpression in zebrafish results in a cleft between the lateral elements of the ethmoid plate, a structural analog of the amniote palate that is found in higher vertebrates, while underexpression results in an abnormal shape of this plate (Eberhart et al., 2008; Dougherty et al., 2012). In this respect, it is interesting to note that miR-140 null mice exhibit shorter palatal bones but no overt cleft palate (Miyaki et al., 2010), which mirrors the phenotype seen in zebrafish. The zebrafish studies have also shown that miR-140 specifically targets *pdgfra* translation, which in turn represses Pdgfa-mediated attraction of both rostrally and caudally migrating anterior cNC cells to the palatal ectoderm. The precise expression level of miR-140 is critical as overexpression will decrease Pdgfa-mediated attraction of both subsets of cNC cells while underexpression inhibits only the rostrally migrating cNC cells to move past the optic stalk. It still remains to be determined whether the same mechanism contributes to the cleft palate in *Pdgfra/Pdgfa* null mice and is associated with *PDGFRA* mutations in humans (Smith and Tallquist, 2010). As the molecular mechanisms that guide cNC cell migration and differentiation are highly conserved in most vertebrates, a similar mechanism is plausible. However, it is important to remember that miR-140 expression increases and is maintained in the developing PS up to and including the fusion of the secondary palate. As zebrafish do not have a nasopharynx, secondary palate formation does not occur and, therefore, it is possible that miR-140 plays an additional role in secondary palate formation among higher vertebrates.

Recent genetic studies have shown that miR-140 is also involved in the etiology of cleft palate in humans. First, a genetic association study showed that a SNP (rs7205289:C>A) located in the precursor of miR-140 (pre-mir-140) contributes to non-syndromic cleft palate susceptibility by influencing the processing of miR-140 (Li et al., 2010). The minor, A allele of rs7205289, with a higher frequency in patients, was associated with a decrease of miR-140-5p expression and an increase of miR-140-3p expression. In addition, miR-140 was found to be down-regulated in palatal mesenchymal cells by smoking. Moreover, an epidemiological analysis revealed that infants with CA/AA genotypes of rs7205289 that were exposed to maternal passive smoking during pregnancy had a higher risk of developing cleft palate (Li et al., 2011). As already mentioned above, *PDGFRA* mutations have been identified in patients with non-syndromic cleft palate. A single base-pair substitution in the 3′UTR of *PDGFRA* was identified that is located only 10 base-pairs away from a predicted binding site for mir-140 (Rattanasopha et al., 2012). Furthermore, this variant is highly conserved in primates and functionally relevant. Genetic evidence thus supports the role of miR-140 dysregulation in the etiology of cleft palate.

#### miR-17-92 cluster as regulator of shelf outgrowth

Palatal shelf outgrowth is an essential step during palatogenesis (Figure 1). During this phase, the shelves increase in size through mesenchymal cell proliferation and the production of extracellular matrix components such as collagen. The mir-17-92 cluster, firstly identified as an inducer of tumor formation through its pro-proliferative effect, has been shown to play a similar role during palatogenesis in mice (Wang et al., 2013). The mir-17-92 cluster is located in the third intron of a ~7 kb primary transcript known as *C13orf25* on human chromosome 13q31.3. It contains 6 miRNAs (miR-17, miR-18a, miR-19a, miR-20a, miR-19b-1, and miR-92a-1), with highly conserved sequences and organization. Ancient genetic duplications have given rise to two miR-17-92 cluster paralogs in mammals: the miR-106b-25 cluster (located on human chromosome 7) and the miR-106a-363 cluster (located on the X chromosome). The expression of mir-17-92 and its 2 paralogs follows a similar pattern in mouse embryos decreasing from E12 to E14 and concentrating in the distal tips of the PS during palatogenesis (Mukhopadhyay et al., 2010; Li et al., 2012; Wang et al., 2013). MiR-106b-25 is expressed at a lower level than miR-17-92.

Homozygous deletion of miR-17-92 in mice leads to perinatal death due to severe hypoplastic lungs and ventricular septal defects (Ventura et al., 2008). As demonstrated by Wang et al., these embryos also have a smaller body size, microcephaly, micropthalmia, mandibular hypoplasia, and an incompletely penetrant cleft palate. This phenotype is similar to that seen in patients with a specific germline deletion of the miR-17-92 cluster (de Pontual et al., 2011). Whereas deletion of the paralogs alone induced no gross abnormalities in mice, compound loss of miR-106b-25 with miR-17-92 leads to a completely penetrant cleft palate (Wang et al., 2013). In addition, the miR-17-92 cluster was shown to regulate osteoblast proliferation and differentiation, with loss of cluster function being associated with bone deficiencies (Zhou et al., 2014). Although no mention is made of a submucous cleft palate in the mouse embryos, it is possible that such a cleft is present, similar to *Tbx22* null mice (Pauws et al., 2009), due to reduced palatal bone formation. Wang et al. found greatly reduced cell proliferation in the PS with aberrant expression of T-box transcription factors and FGF signaling, both targets of this cluster. It was also identified that the expression of miR-17-92 is regulated through BMP signaling, a deficiency of which was shown to cause cleft palate and other craniofacial anomalies. In addition, the master regulator of cranial neural crest development AP-2a is involved in the regulation of miR-17-92 (Wang et al., 2013). Interestingly, miR-92a also maintains BMP signaling during pharyngeal cartilage formation (Ning et al., 2013), suggesting a positive feedback loop between the miR-17-92 cluster and BMP signaling. In addition, a functional synergy has been identified between the miR-17-92 cluster and the SHH signaling pathway, which itself drives palatal shelf outgrowth and functionally interacts with the BMP signaling pathway (Uziel et al., 2009; Greene and Pisano, 2010).

Most importantly, overexpression of the miR-17-92 cluster within palatal mesenchymal cells results in increased proliferation by inhibiting the normal TGF-β signaling pathway (Li et al., 2012). This corroborates the above in vivo studies. However, collagen synthesis was also decreased in these cells. *In vivo* and *in vitro* studies thus suggest that the miR-17-92 cluster controls palatogenesis by targeting several regulators of cell proliferation, analogous to its effect in cancer development. In addition, this cluster affects collagen synthesis, which also plays an essential role during palatal shelf outgrowth.

#### miR-200b as regulator of palatal fusion

In the last phase of palatogenesis, the epithelium between the two contacted palatal shelves—the midline epithelial seam (MES)—needs to be removed to provide mesenchymal continuity. The disintegration of the MES is likely due to three mechanisms, namely epithelial-to-mesenchymal transition, cell death and migration of the MES cells (Bush and Jiang, 2012). MiR-200b belongs to the miR-200 family and together with other family members miR-200a and miR-429, it is clustered in an intergenic region on human chromosome 1.

MiR-200b is expressed in the epithelium during palatogenesis in the mouse, including in the midline epithelial seam (MES), and its expression gradually decreases as fusion proceeds (Shin et al., 2012a,b). In keeping with this, overexpression of miR-200b results in a failure of fusion due to persistence of the MES (Shin et al., 2012a,b). In this respect, miR-200b was shown to target *Smad2, Snail, Zeb1*, and *Zeb2*, all genes encoding transcription factors that function as mediators of the Tgf-β signaling pathway. In response to TGF-β, SMAD2/3 is activated, forms a complex with SMAD4, which then interact with either ZEB1, ZEB2, or SNAIL to repress epithelial markers, stimulate mesenchymal markers, and induce migration and apoptosis. Overexpression of miR-200b also leads to maintenance of the MES by repressing TGF-β during the final stages of palatogenesis and hence results in a failure of the PS to fuse.

#### Conclusions and future perspectives

The processes of palatal shelf growth, elevation and fusion require precise spatiotemporal gene expression patterns. This is also reflected by the critical role of transcription factors in palatogenesis. Advances in genomics have made it clear that certain non-coding regions of the genome are predominant gene regulators. MiRNAs are small non-coding RNAs that function as post-transcriptional repressors. They are essential for embryonic development, and depletion of miRNAs in the mesenchyme and oral ectoderm of mouse embryos leads to cleft palate. With the exponential increase in new miRNAs being identified, it is likely that many miRNAs will turn out to have a role during palatogenesis. However, to date, the role of only a few miRNAs in palatogenesis has been established in mice (Table 1). In summary, miR-140 regulates the migration of neural crest cells, miR-200b regulates palatal fusion and the miR-17-92b cluster regulates palatal shelf growth. Genomic studies of miRNAs in nsCL/P and nsCPO are only just starting. Two studies from the same research group analyzed miRNA expression in plasma of nsCL and nsCL/P patients (Li J. et al., 2016; Zou et al., 2016). Using a microarray screening method, 305 miRNAs in plasma of nsCL patients and 241 miRNAs in plasma of nsCL/P patients were found to be differentially expressed compared with healthy controls. As miRNAs have a tissue-specific expression and role it is, however, more interesting to study their expression in the relevant tissues. Another study on non-syndromic cleft lip/palate identified a SNP in the 3′UTR of *MSX1* that resulted in an altered target recognition by miR-3649 and a differential expression between cases and controls (Ma et al., 2014). Similarly, altered miR-496-FGF2, miR-145-FGF5, and miR-187-FGF9 interactions were associated with clefting in 289 nsCLP and 49 nsCPO patients (Li D. et al., 2016). This provides further proof that polymorphisms in miRNAs and their target sites are sources of phenotypic variation. Therefore, future studies on miRNA polymorphisms and cleft palate may provide a good basis for increasing our knowledge about the genetic risk variants contributing to non-syndromic cleft palate.

**Table 1:** **miRNAs genes or targeted mRNAs which have been associated or causally linked with cleft palate in humans or mice and cleft ethmoid plate in zebrafish**.

**miRNA gene or target mRNA**	**Species**	**Genome variation**	**Molecular effect**	**References**
*PDGFRa*	Human	Mutation 3′UTR	Altered miR-140 binding	Rattanasopha et al., 2012
*miR-140*	Human	SNP	Altered miRNA-140 processing	Li et al., 2010, 2011
	Zebrafish	Overexpression	Altred Pdfra repression	Eberhart et al., 2008
*MSX1*	Human	SNP 3'UTR	Altered miR-3649 binding	Ma et al., 2014
*FGF2/5/9*	Human	SNP3'UTR	Altered miR-496/miR-145/miR-187 binding	Li D. et al., 2016
*miR-17-92 cluster*	Mouse	Homozygous deletion	Altered Tbx113, Fgf10, Shox2 & Osr1 repression	Wang et al., 2013
*miR-200b*	Mouse	Overexpression	Altered Smad2, Snail& Zeb112 repression	Shin et al., 2012a,b
*miR-133b*	Zebrafish	Overexpression	Unkown	Ding et al., 2016

## Author contributions

CS, CC, JV: Conception of the work, drafting of the manuscipt, revision of the manuscript, final approval of the manuscript. AA, MT, GP: Conception of the work, Revision of the manuscript, final approval of the manuscript.

### Conflict of interest statement

The authors declare that the research was conducted in the absence of any commercial or financial relationships that could be construed as a potential conflict of interest.
